# Supplementary material for: Distinct cytoskeletal proteins define zones of enhanced cell wall synthesis in Helicobacter pylori
Source: eLife. 2020 Jan 9;9:e52482. doi: 10.7554/eLife.52482 (PMC7012605; doi:10.7554/eLife.52482)
Supplement: Transparent reporting form [file elife-52482-transrepform.pdf]

eLIFE
1st Floor
24 Hills Road
Cambridge CB2 1JP, UK
P 01223 855340
W elifesciences.org
T @elife

eLife's transparent reporting form

We encourage authors to provide detailed information *within their submission* to facilitate the interpretation and replication of experiments. Authors can upload supporting documentation to indicate the use of appropriate reporting guidelines for health-related research (see EQUATOR Network), life science research (see the BioSharing Information Resource), or the ARRIVE guidelines for reporting work involving animal research. Where applicable, authors should refer to any relevant reporting standards documents in this form.

If you have any questions, please consult our Journal Policies and/or contact us: editorial@elifesciences.org.

Sample-size estimation

- You should state whether an appropriate sample size was computed when the study was being designed
- You should state the statistical method of sample size computation and any required assumptions
- If no explicit power analysis was used, you should describe how you decided what sample (replicate) size (number) to use

Please outline where this information can be found within the submission (e.g., sections or figure legends), or explain why this information doesn't apply to your submission:

The number of cells used for analysis is shown in the figure and/or is stated in the legend (Figure 2, 6, 7, 8, 10, 11 and associated supplements). Based on cell density within fields of view for each sample, we aimed to collect enough images to yield at least 100 cell reconstructions for each biological replicate of each condition. We performed and pooled three biological replicates for each main figure curvature enrichment plot. As shown in the corresponding figure supplements, individual biological replicates are reproducible.

Replicates

- You should report how often each experiment was performed
- You should include a definition of biological versus technical replication
- The data obtained should be provided and sufficient information should be provided to indicate the number of independent biological and/or technical replicates
- If you encountered any outliers, you should describe how these were handled
- Criteria for exclusion/inclusion of data should be clearly stated
- High-throughput sequence data should be uploaded before submission, with a private link for reviewers provided (these are available from both GEO and ArrayExpress)

Please outline where this information can be found within the submission (e.g., sections or figure legends), or explain why this information doesn't apply to your submission:

The number and type of replicates used is reported in each figure legend and the definition of the replicates is defined for each experiment in the methods section.

eLife Sciences Publications, Ltd is a limited liability non-profit non-stock corporation incorporated in the State of Delaware, USA, with company number 5030732, and is registered in the UK with company number FC030576 and branch number BR015634 at the address 1st Floor, 24 Hills Road, Cambridge CB2 1JP | August 2014

Statistical reporting

- Statistical analysis methods should be described and justified
- Raw data should be presented in figures whenever informative to do so (typically when N per group is less than 10)
- For each experiment, you should identify the statistical tests used, exact values of N, definitions of center, methods of multiple test correction, and dispersion and precision measures (e.g., mean, median, SD, SEM, confidence intervals; and, for the major substantive results, a measure of effect size (e.g., Pearson's r, Cohen's d)
- Report exact p-values wherever possible alongside the summary statistics and 95% confidence intervals. These should be reported for all key questions and not only when the p-value is less than 0.05.

Please outline where this information can be found within the submission (e.g., sections or figure legends), or explain why this information doesn't apply to your submission:

Applicable statistical analysis is reported in the figure legends (Figure 3, 6, 7, 10, 11 and associated supplements), associated methods section ("3D reconstructions and curvature enrichment"), and Appendix 1.

(For large datasets, or papers with a very large number of statistical tests, you may upload a single table file with tests, Ns, etc., with reference to sections in the manuscript.)

Group allocation

- Indicate how samples were allocated into experimental groups (in the case of clinical studies, please specify allocation to treatment method); if randomization was used, please also state if restricted randomization was applied
- Indicate if masking was used during group allocation, data collection and/or data analysis

Please outline where this information can be found within the submission (e.g., sections or figure legends), or explain why this information doesn't apply to your submission:

Group allocation does not apply to our submission as all experiments are performed on bacterial strains in broth culture or on agar plates. For SIM imaging and subsequent analysis, the same set of sampling criteria and analysis parameters were used for all conditions.

Additional data files ("source data")

- We encourage you to upload relevant additional data files, such as numerical data that are represented as a graph in a figure, or as a summary table
- Where provided, these should be in the most useful format, and they can be uploaded as "Source data" files linked to a main figure or table
- Include model definition files including the full list of parameters used
- Include code used for data analysis (e.g., R, MatLab)
- Avoid stating that data files are "available upon request"

Please indicate the figures or tables for which source data files have been provided:

eLife Sciences Publications, Ltd is a limited liability non-profit non-stock corporation incorporated in the State of Delaware, USA, with company number 5030732, and is registered in the UK with company number FC030576 and branch number BR015634 at the address 1st Floor, 24 Hills Road, Cambridge CB2 1JP | August 2014

The MATLAB scripts used to reconstruct cell surfaces, perform the geometric enrichment analyses, and fit 3D helical centerlines are publicly available under a BSD 3-clause license at https://github.com/PrincetonUniversity/shae-cellshape-public and archived at https://doi.org/10.5281/zenodo.1248978. The individual 3D cell surface reconstructions are stored as .mat MATLAB data files and can be interacted with using the tools described above. Images, MATLAB reconstructions, and sequence data will be archived by Princeton University and will be publicly accessible.
